# Adsorption of methylene blue onto electrospun nanofibrous membranes of polylactic acid and polyacrylonitrile coated with chloride doped polyaniline

**DOI:** 10.1038/s41598-020-69825-y

**Published:** 2020-08-07

**Authors:** Noor Mohammad, Yomen Atassi

**Affiliations:** Department of Applied Physics, Higher Institute for Applied Sciences and Technology, Damascus, Syria

**Keywords:** Environmental sciences, Natural hazards, Chemistry, Materials science, Nanoscience and technology

## Abstract

This study presents the preparation of membranes of polylactic acid (PLLA), polyacrylonitrile (PAN) and their corresponding membranes coated with polyaniline (PANI) for the adsorption of methylene blue (MB). Scanning electron microscopy micrographs reveal that all the membranes exhibit nanofibrous morphology. The adsorption capacity and the removal efficiency of the membranes are studied as a function of (initial adsorbate concentration, pH of the medium, temperature, contact time and adsorbent dosage). Coated membranes with PANI showed better adsorption performance and their DC conductivities were correlated to MB concentrations. Adsorption isotherms have also been performed and the adsorption process has been tested according to Langmuir and Freundlich models. The regeneration and reuse of the prepared membranes to re-adsorb MB were also investigated. The enhancement in adsorption performance and reusability of PANI-coated membranes in comparison with non-coated ones are fully discussed.

## Introduction

Dyes are indispensable materials in many industrial fields such as textiles, food, cosmetics, paper, rubber and plastics^1,2^. The industrial use of these dyes poses a potential threat to the aquatic ecosystems, due to the ease of dyes transfer into waterways^3^. The most important risk of industrial dyes is linked to their resistance to environmental conditions, which makes their biological degradation a difficult process^3,4^. In addition, the products of degradation for some dyes also have either potential toxic or mutagenic and carcinogenic effects which result in additional pollution to the ecosystem^5,6^. The color resulted from dyes in effluents also plays a critical contaminant role towards aquatic ecosystems^7^. The color of contaminant dyes impedes the growth of bacteria and hinders the photosynthesis by absorbing sunlight entering the ecosystem. The hazardous chronic effects of dyes on organisms differ according to the concentration of the dye and time of exposure^7–9^. Some studies show that even a very low concentration of the contaminant (as low as 0.005 ppm) could have a significant danger on organisms^9,10^.

Due to dyes hazardous effects, their removal from water and the remediation of contaminated water have received increasing attention and extensive research has been performed in this specific field of environmental science^6^. In this respect, a myriad of techniques has been investigated such as chemical oxidation, adsorption, coagulation, precipitation, to name a few^3,11,12^. Among those methods, adsorption has been interestingly studied because of its advantages including low costs, simplicity of application, effectiveness and possibility of adsorbent reuse and regeneration^13–16^. Furthermore, another important advantage of the adsorption method is the possibility of adsorbent surface modification, which allows the enhancement of adsorbent performance^3,13^. Several types of adsorbents have been developed with the objective to obtain more removal efficiency, better selectivity and longer lifetime^14^. The researchers were particularly interested in developing cheaper adsorbents from some materials such as teakwood, clay, rice husk, orange peel, activated sludge, etc.^14–18^. These materials have remained quite unsatisfactory in their performance for removing the dyes with the required effectiveness because they lead to producing large amounts of sludge and as a result causes additional pollution^3,15^.

Recently, a new type of adsorbents based on inherently conducting polymers (ICPs) has emerged^3,19,20^. ICPs exhibit unique physical and chemical properties that attracted researchers’ attention to use them as adsorbents^12^. Polyaniline (PANI) is one of the most studied ICPs. It is a poly aromatic amine that can be readily synthesized by the oxidative polymerization^21^. Bulk PANI has shown effective adsorption toward dyes such as azo dyes owing to its chemical structure that includes amine and imine group^22^.

To enhance dye removal by PANI, membrane technology is the method of choice as it offers a high surface to volume ratio^23^. Therefore, a PANI-membrane was prepared by casting freestanding films from PANI dispersions or by casting PANI blends dispersions^23^. However, electrospinning is another powerful technique to prepare polymer membranes. The key feature of this technique for membrane technology resides in its potentiality to control pore sizes and get high specific surface area^23^. Electrospinning allows the drawing of fibers with diameters of about some hundred nanometers under applied electric force on polymer solutions or polymer melts^24,25^. The electrospun membrane does not only exhibit enhanced adsorption, but also has good filtration characteristics^26^. The use of PANI-based membranes prepared by electrospinning technique, to treat wastewater, is a new trend and there are few studies on that. Alcaraz-Espinoza et al. deposited PANI on electrospun polystyrene membranes for water remediation by adsorption of heavy metal ions on the prepared membrane^27^. Aziz et al., prepared an electrospun multilayered composite membrane of silk fibroin/PAN with different ratio of PANI/TiO_2_ nanoparticles, which finally was used as a filter for anionic HFGR black dye^28^. In a recent work, published by our team, novel electrospun nanofibrous membranes, of polylactic acid and coated with pTSA-PANI, were used for the remediation of wastewater of anionic pollutants^29^.

In this study, two types of PANI-based membranes prepared by the electrospinning technique were investigated for the treatment of wastewater polluted with cationic organic pollutants. In this respect, methylene blue dye (MB) is used as an example of cationic organic dyes. Firstly, membranes of polylactic acid (PLLA) and polyacrylonitrile (PAN) were prepared each from their own solutions, using the electrospinning technique. Then, PLLA and PAN membranes were coated with chloride doped polyaniline PANI using the in-situ oxidative polymerization method, PLLA/PANI and PAN/PANI, respectively. The removal efficiency and the adsorption capacity of the membranes were examined towards methylene blue. The effects of MB concentration, contact time, temperature, pH of the solution and adsorbent dosage on the adsorption capacity and removal efficiency of the membranes were investigated. Also, adsorption kinetics and adsorption isotherms were studied. Electrical characterization of the membranes was also performed and the correlation between the electrical conductivity and MB concentration was reported. The enhancement in adsorption performance and regeneration ability of PANI-coated membranes were fully investigated. To the best of the authors’ knowledge, the use of PLLA and PAN nanofibrous membranes coated with PANI for water remediation of cationic pollutants has not been reported before.

## Experimental

### Materials

Polylactic acid (Mw 105,000) from Nature work, polyacrylonitrile (Mw 120,000), tetra n-butyl ammonium bromide (TBAB), anilinium chloride (≥ 99%), Dichloromethane (DCM), dimethylformamide (DMF) and methylene blue all were purchased from Sigma-Aldrich and used as received without purification. All other reagents were analytical and also used without any further purification.

### Instruments and methods

Programmable syringe pump (TOP-5300, Japan) was used to prepare the electrospun membranes. Morphology studies of the prepared membranes were conducted by scanning electron microscope (SEM) using Tescan Vega-II XMU SEM. FTIR spectra were recorded by BRUKERVECTOR22 FTIR spectrophotometer. UV–Vis spectra were recorded using UV–Vis spectrometer (JASCO, V-350). pH measurements were performed by (TWT, pH7110) pH meter. The DC-conductivity $(\sigma )$ of the membranes was measured using the four-probe technique. To perform the measurement of $(\sigma )$ a homemade four-probe device, composed of four parallel platinum wires of 0.4-mm diameter positioned 2 mm of each other, was used. KEITHELY-220 and KEITHELY-617 were used as a programmable current source and programmable electrometer, respectively. $\sigma$ is calculated using the Van der Pauw equation $\sigma = \frac{d}{t \cdot w}\frac{I}{V}$, where $d$ is the distance between the electrodes, $t$ and $w$ are the sample’s thickness and width respectively. Wettability tests were performed using a digital camera Promate for measuring the contact angle.

For the adsorption tests, 10 mg of the samples were immersed in 10 mL of MB solution (i.e. adsorbent dosage 1 g/L) for 24 h to make sure that the adsorption process reaches the equilibrium ^30^. The concentration of MB in the solution was determined by measuring UV–Vis absorbance at 665 nm wavelength which represents the maximum absorption wavelength of methylene blue. Adsorption capacity $q$ and removal efficiency $R\%$ are calculated using the following equations^30^:

1 $$ q = \frac{{(C_{0} - C_{e} )v}}{m} $$

2 $$ R\% = \frac{{(C_{0} - C_{e} )}}{{C_{0} }} \times 100 $$

where $C_{0}$ is the initial dye concentration, $C_{e}$ is the equilibrium dye concentration, $v$ is the volume of the dye solution and $m$ is the mass of the used membrane.

The specific surface area ($S$) of the prepared membranes were calculated as follow: $S = {A \mathord{\left/ {\vphantom {A m}} \right. \kern-\nulldelimiterspace} m}$ where $A$ is the total surface area and $m$ is the mass of membrane. Using the assumption that the nanofibers are cylinders with definite length $A = {{4m} \mathord{\left/ {\vphantom {{4m} {(\rho \cdot D)}}} \right. \kern-\nulldelimiterspace} {(\rho \cdot D)}}$^30^. Where $D$ is the mean fiber diameter and $\rho$ is the fiber density measured based on Archimedes method. Hence, the equation for calculating the specific surface area becomes:

3 $$ S = {4 \mathord{\left/ {\vphantom {4 {(\rho \cdot D)}}} \right. \kern-\nulldelimiterspace} {(\rho \cdot D)}} $$

Also, the membrane porosity $P\%$ was calculated using the equation^30,31^:

4 $$ P\% = \frac{{(V_{app} - V_{{}} )}}{{V_{app} }} \times 100 $$

where $V_{app}$ is the apparent volume estimated by measuring the thickness and the surface area of the membrane and $V_{{}}$ is the theoretical volume estimated from the membrane mass and bulk density.

### Preparation of PLLA and PAN membranes

The PLLA and PAN membranes were prepared by electrospinning starting from homogenous solutions as reported elsewhere^32^, as follows: PLLA electrospinning solution was obtained by dissolving PLLA granules in DCM (6% w/w) under magnetic stirring for 6 h. Then, 1 wt% of the TBAB is added to the polymer solution during stirring to enhance the electrical conductivity. While the conductivity value of the PLLA solution is only 2 μS cm^−1^, the conductivity of its solution with 1% TBAB is 380 μS cm^−1^. In the next step, the polymer solution was placed in a 5 mL syringe with a hollow metal needle (nozzle) which has an inner diameter of 0.6 mm and outer diameter of 0.8 mm. The flow rate of the polymer solution was fixed at 1 mL h^−1^, and the distance between the injector head and the flat collector was set at 13 cm.

When the value of the applied voltage reaches 20 kV, the electric force generated by the electric field can overcome the surface tension of the polymer solution and then Taylor cone forms and the nanofibers begin to move from the Taylor cone head towards the collector and place smoothly on the collector.

In all electrospinning processes, the value of applied voltage was kept between 19 and 22 kV to ensure the stability of Taylor cone and the electrospinning process. In addition, the temperature in the electrospinning chamber was kept at 22 ± 2 °C and the humidity in the range between 40 and 50% to ensure good evaporation of the solvent from the fiber during its movement towards the collector. The electrospinning process was continued for 1 h. The resulted nanofibrous membranes deposited on the collector were then left for 2 h in the spinning chamber. Finally, the nanofibrous membrane was stripped off and kept in dark for use in subsequent experiments.

The same procedure was used for PAN membrane preparation from polymer solution in DMF (4.5% w/w) and using the following parameters: the distance between the injector head and the flat collector was 20 cm, the electrospinning time was 30 min and the value of applied voltage was 15 kV.

### Preparation of PLLA/PANI and PAN/PANI membranes

PLLA membranes have circular shape with 15 cm diameter and 100 μm thickness. The PLLA/PANI nanocomposite membranes are prepared using the in situ chemical oxidative polymerization method. To prepare the composite membrane, 1.295 g (10 mmol) of anilinium chloride is dissolved in 25 mL of distilled water aided by ultra-sonication, and 2.855 g (12.5 mmol) of ammonium persulfate (APS) is dissolved in 25 mL of distilled water using magnetic stirring. Both solutions are left under stirring for half an hour at room temperature (~ 20 → 24 °C). Then, the PLLA membrane is immersed in the prepared anilinium chloride solution for 30 min to ensure that the membrane is impregnated well in the solution. The APS solution is then dropped on the membrane for 30 min. Aniline monomer was oxidized by APS and was doped by chlorine ions. The previous mixture is left for 3 h to ensure that all aniline monomers are involved in the polymerization reaction and to ensure that the reaction is completely finished. After that, the membrane is washed many times with distilled water and then with acetone until a colorless effluent is obtained. The membrane is then washed three times using 50 mL of 0.2 M HCl solution and three times with acetone to remove all byproducts of the polymerization reaction. The membrane is finally placed in a drying oven at 40 °C for 24 h, and then stored in a dark closed container.

**Figure 1:**
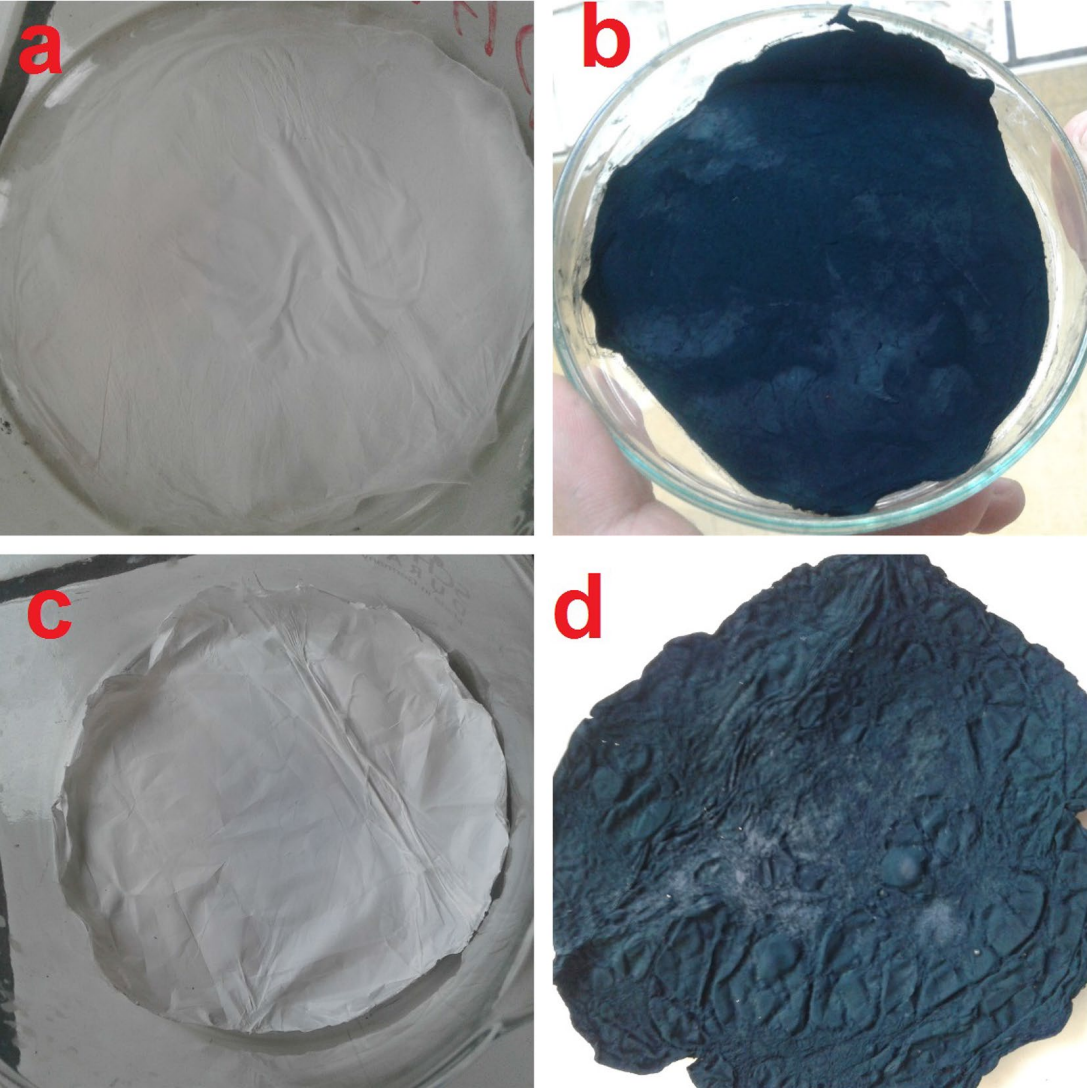
Digital photos of the four membranes, (**a**) PAN; (**b**) PAN/PANI; (**c**) PLLA; (**d**) PLLA/PANI.

The same procedure was conducted for PAN to obtain PAN/PANI nanocomposite membranes. Digital photos of PAN, PAN/PANI, PLLA and PLLA/PANI membranes are shown in Fig. 1.

### Informed consent

No human studies were carried out by the authors for this article. No animal studies were carried out by the authors for this article.

## Results and discussion

### Morphology characterization

**Figure 2:**
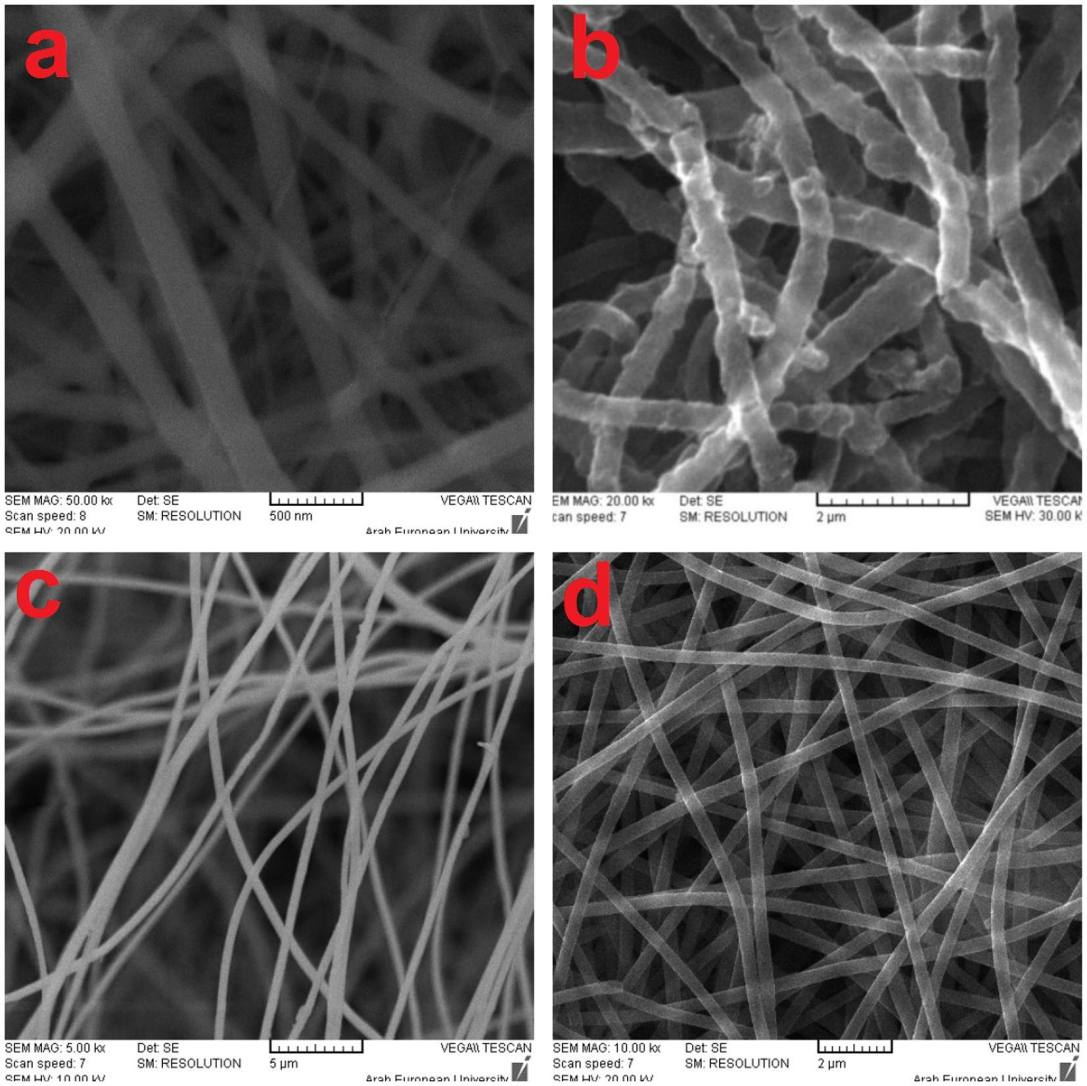
SEM micrographs of the four membranes, (**a**) PLLA, (**b**) PLLA/PANI, (**c**) PAN and (**d**) PAN/PANI.

The prepared membranes were characterized by scanning electron microscope (SEM) to investigate the nanostructure and to understand its effect on the adsorption performance. The SEM images in Fig. 2 shows that PLLA membrane has fibrous structure with 186 nm fiber diameter, and the fibers are long with fine and smooth surface. The PLLA/PANI membranes exhibit rougher structure with diameter of 518 nm. PAN membrane micrograph shows that the fibers are beads free and they are continuous with a diameter of 330 nm. PAN/PANI membrane has smooth nanofibers as PAN membrane, but with higher diameter of about 418 nm owing to PANI deposition. The above-mentioned results indicate that PANI has successfully covered the whole PAN surface.

The estimation of membrane specific surface area and porosity was performed using Eqs. 3 and 4, and was summarized in Table 1 along with the estimated porosity from SEM micrographs $P_{SEM} \%$ using ImageJ software^33^. From Table 1, it is observed that PLLA, PAN nanofibrous membranes have higher specific surface area and higher porosity than PLLA/PANI and PAN/PANI membranes, respectively. The decrease in specific area when depositing PANI on membranes could be linked to diameter increase due to the deposition of PANI coating. One should notice that the estimated porosity and the one calculated based on Eq. (3) are almost equal and the results are correlated. The porosity slightly decreases upon coating.

**Table 1:** Specific surface area and porosity [calculated from Eq. (4) and estimated from SEM micrographs] of the prepared membranes.

Sample	$S\left( {{{{\text{m}}^{2} } \mathord{\left/ {\vphantom {{{\text{m}}^{2} } {\text{g}}}} \right. \kern-\nulldelimiterspace} {\text{g}}}} \right)$	$P\%$	$P_{SEM} \%$
PLLA	12.1 ± 0.2	89 ± 3	82 ± 3
PLLA/PANI	7.0 ± 0.4	85 ± 2	80 ± 1
PAN	15.1 ± 0.2	82 ± 2	79 ± 2
PAN/PANI	10.0 ± 0.3	77 ± 2	74 ± 2

### FTIR spectra

**Figure 3:**
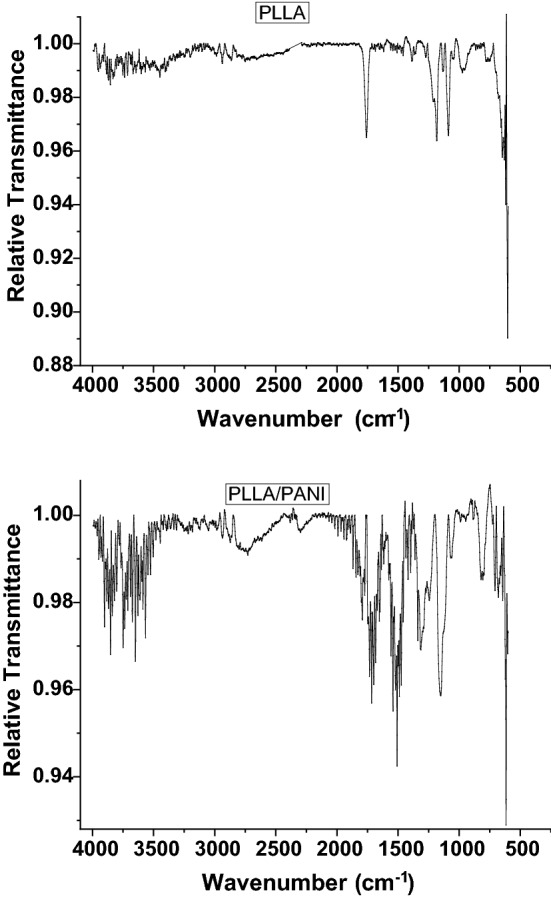
FT-IR spectra of PLLA nanofibers membrane and PLLA/PANI composite nanofibers membrane.

FTIR spectra of PLLA and PLLA/PANI membranes are exhibited in Fig. 3, whereas their main characteristic peaks are listed in Table 2. It can be readily seen that the main peaks of PLLA were located at 3,448, 2,939, 1,754, 1,460, 1,385, 1,080, 1,038, 865 cm^−1^^32^. In the comparison between PLLA and PLLA/PANI spectra, one can easily note that the peak for the O–H group has a shift to lower wavenumbers when PLLA coated with PANI. This also applies to carbonyl group C=O that shifts from 1754 to 1715 cm^−1^ in the PLLA/PANI membrane. This observation suggests the intermolecular bonding between PLLA and and PANI chains. Moreover, the presence of new peaks in the membrane spectrum after coating with PANI, such as the C=C aromatic in the benzene ring at 1,468 cm^−1^ and 1544 cm^−1^, and the appearance of the C–N stretching vibration peak at 1,305 cm^−1^ and the N–H stretching peak at 3,440 cm^−1^, suggests the successful coating of PLLA membrane with PANI.

**Table 2:** Main characteristic peaks of PLLA and PLLA/PANI.

PLLA/PANI (cm^−1^)	PLLA (cm^−1^)	Bond vibration
3,424	3,448	O–H stretching
2,936	2,939	C–H stretching
1715	1754	C=O stretching
1,468	–	C=C stretching in benzene ring
1544		
1,305	–	C–N stretching
3,440	–	N–H bending
1,156	1,190 as	COC stretching
1,059	1,080 s	
815	–	C–H out of plane bending
1,377	1,385	CH_3_ s
1,453	1,460	CH_3_ as
1,031	1,038	C–CH_3_
814	865	OCC

**Figure 4:**
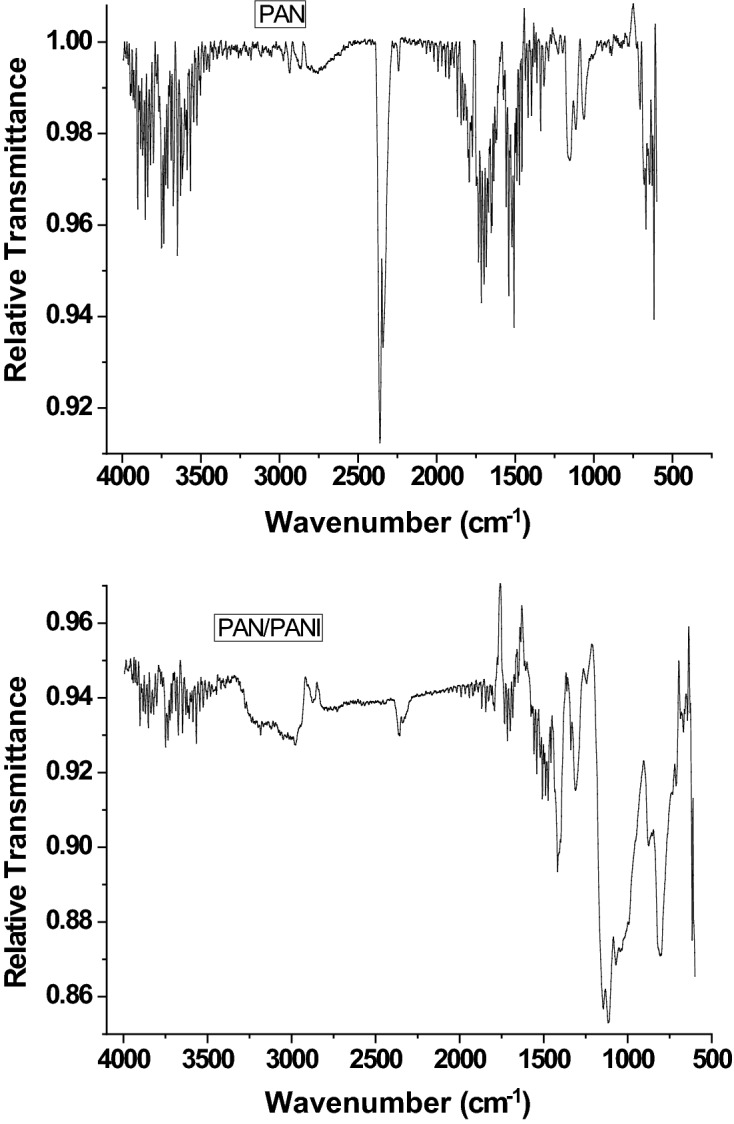
FT-IR spectra of PAN nanofibrous membrane and PAN/PANI composite nanofibers membrane.

FTIR spectra of PAN and PAN/PANI membranes are exhibited in Fig. 4, whereas their main characteristic peaks are listed in Table 3.

**Table 3:** Main characteristic peaks of PAN and PAN/PANI.

PAN/PANI (cm^−1^)	PAN (cm^−1^)	Bond
1,421	1,470	C–H scissoring
2,884	2,950	C–H stretching
2,240	2,252	C≡N
1,483	–	C=C stretching in benzene ring
1,640		
1,312	–	C–N stretching
3,510	–	N–H stretching
3,090	–	=C–H stretching

PAN spectrum reveals distinct peaks corresponding to the stretching of the triple bond between carbon and nitrogen, C–H scissoring and C–H stretching at 2,252, 1,470, 2,950 cm^−1^, respectively. When the PANI is deposited on the PAN, new peaks appear such as the C=C aromatic stretching at 1,483 cm^−1^ and 1,640 cm^−1^, as well as the C–N stretching at 1,312 cm^−1^, the N–H stretching at 3,510 cm^−1^ and =C–H stretching with sp^2^ hybridization within the aromatic ring. The triple bond between the carbon and nitrogen has a red shift and its intensity has markedly decreased, confirming the interaction between PAN as membrane and PANI and suggesting the successful deposition of PANI on PAN membrane.

### Membrane wettability

**Figure 5:**
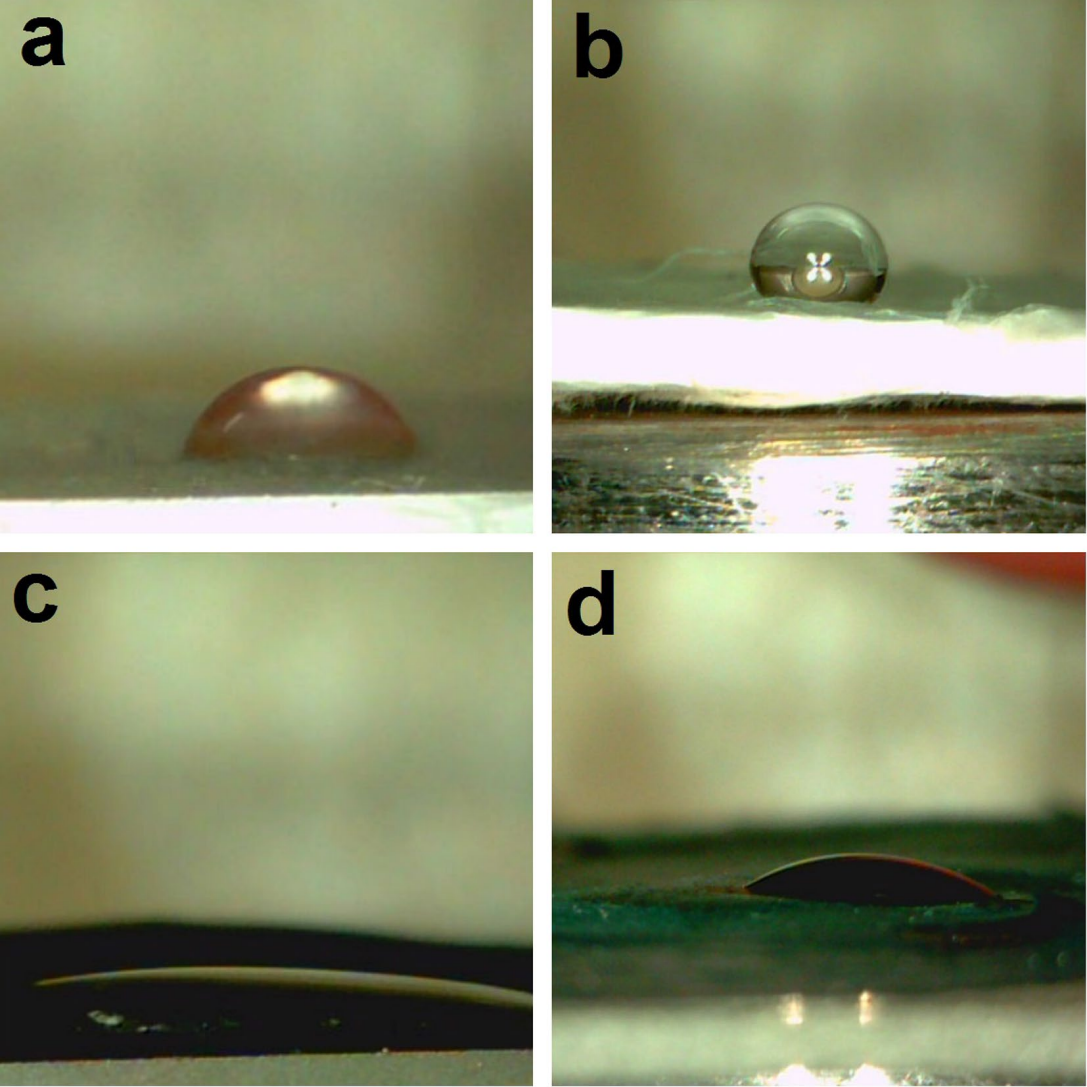
Wettability test for prepared membranes, (**a**) PLLA; (**b**) PAN; (**c**) PLLA/PANI; (**d**) PAN/PANI.

Using the contact angle method, the wettability of the prepared membranes was measured. From Fig. 5 and Table 4, it can be seen that the PLLA/PANI and PAN/PANI membranes showed better wettability compared to the PLLA and PAN membranes, respectively. The contact angle decreases from 70° for PLLA membrane to 18° for PLLA/PANI membrane, whereas the contact angle decreases from 126° for PAN membrane to 34° for PAN/PANI membrane. These results indicate that the coating of the membranes with the PANI is beneficial for increasing the hydrophilicity of the membranes^34^. The increase in the wettability of the membrane enhances the interactions between the adsorbate and the adsorbent.

**Table 4:** Wetting angles of the prepared membranes.

Membrane	Wetting angle (°)
PLLA	70
PLLA/PANI	18
PAN	126
PAN/PANI	34

### Conductivity measurement

The DC-conductivity was measured by four-probe technique by KEITHELY-220 programmable current source and KEITHELY-617 programmable electrometer.

**Figure 6:**
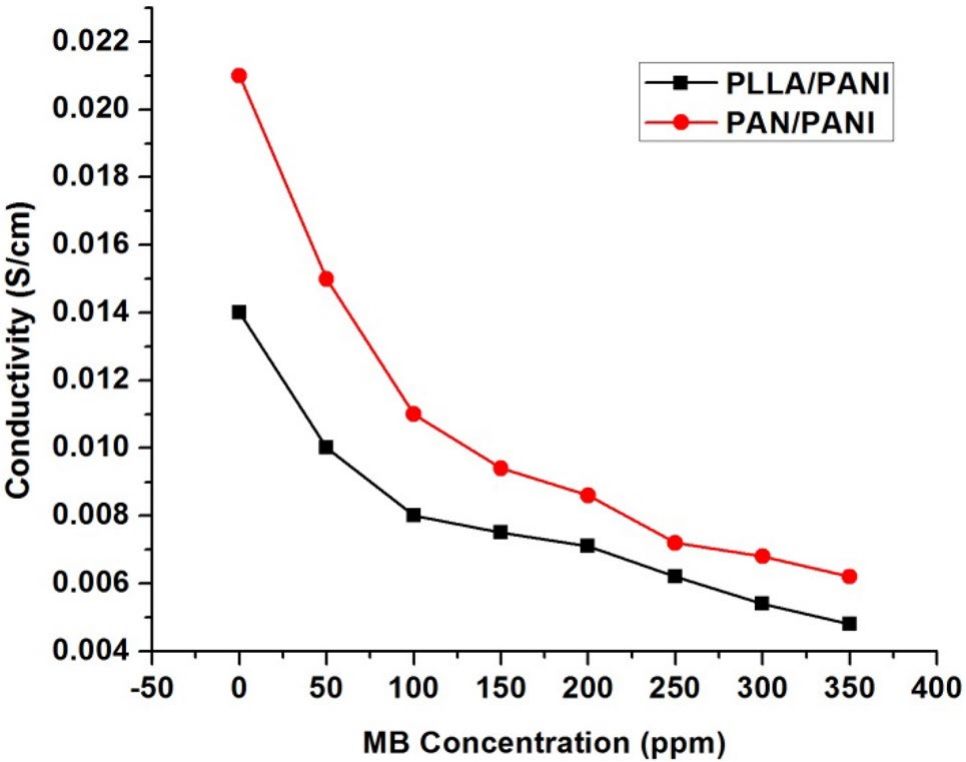
PLLA/PANI and PAN/PANI membranes conductivity after immersing them in MB solutions at different concentrations.

The value of the conductivity was investigated before and after immersion of the prepared membranes with a solution of MB at different concentrations. The PANI is considered as a p-type semiconductor, where conductivity is caused by the movement of conjugated π electrons along the polymeric chain^35^. Before adsorption, PLLA/PANI and PAN/PANI membrane conductivities were 0.014 S cm^−1^ and 0.021 S cm^−1^, respectively. As the concentration of MB increases, the conductivity of the membrane decreases (Fig. 6). The decrease in electrical conductivity with the increase of MB concentration could be linked to the fact that the adsorbate forms a kind of insulating layer on top of the surface of the membrane that decreases its conductivity. It is worthy to note that the correlation between the conductivity of the membrane and MB concentration allows monitoring the pollutant concentration by simple conductivity measurements, especially in the region where the correlation is linear.

### Methylene blue adsorption on the prepared membranes

#### Effect of methylene blue concentration

To investigate the effect of methylene blue concentration on the adsorption properties of the membranes, a series of concentrations was prepared, including 50, 100, 150, 200, 250, 300 and 350 ppm. The membranes were immersed in the prepared solutions for 24 h at room temperature with the adsorbent dosage of (m/v = 1/1 g L^−1^).

**Figure 7:**
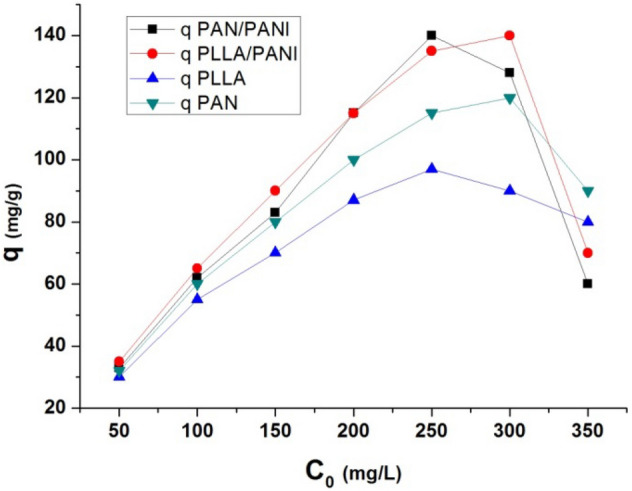
Effect of initial MB concentration on the adsorption capacity.

The maximum adsorption capacity of PLLA and PLLA/PANI membranes was 97 and 135, respectively at concentration of methylene blue of 300 mg L^−1^. On the other hand, the maximum adsorption capacities of PAN, PAN/PANI membranes were 115, 140 mg g^−1^, respectively, at concentration of methylene blue of 250 and 300 mg L^−1^, respectively (Fig. 7).

The increase in adsorption capacity with can be linked to the increase in the concentration gradient which results in higher driving mass to the solid phase^30^.

Then, there is a decrease in adsorption capacity as the initial concentration increases. The decrease in adsorption capacity values after reaching a maximum value was also reported for the first time by Wang et al. when studying the adsorption of MB on wheat straw-derived biochar^36^. They linked this decrease to the fact that MB undergoes hydrolysis in water, and the existing forms in water include both the well accepted $M{B}^{+}$ and the hydrolyzed form of $M{B}^{+}$, (i.e. $M{B}^{+}O{H}^{-}{\left({H}_{2}O\right)}_{n}$)). According to Wang et al., the hydrolysis of MB becomes more obvious when the concentration of MB solution becomes higher, since the pH value of MB solution decreases with the increase of MB concentration. Using Gaussian 09 program, they proved that the interaction energy between $M{B}^{+}O{H}^{-}{\left({H}_{2}O\right)}_{n}$ species is higher than the interaction energy between $M{B}^{+}O{H}^{-}{\left({H}_{2}O\right)}_{n}$ and the adsorbent. This means that the sorbed $M{B}^{+}O{H}^{-}{\left({H}_{2}O\right)}_{n}$ by the adsorbent can be knocked down by the free $M{B}^{+}O{H}^{-}{\left({H}_{2}O\right)}_{n}$ when the concentration of MB is high enough and the free $M{B}^{+}O{H}^{-}{\left({H}_{2}O\right)}_{n}$ has high chance to meet the sorbed $M{B}^{+}O{H}^{-}{\left({H}_{2}O\right)}_{n}$. Moreover, they proved that the volume of $M{B}^{+}O{H}^{-}{\left({H}_{2}O\right)}_{n}$ is higher than the volume of $M{B}^{+}$. This size difference between the two forms of MB in water is good enough to explain why the membranes have obvious larger sorption quantity for $M{B}^{+}$ than for $M{B}^{+}O{H}^{-}{\left({H}_{2}O\right)}_{n}$. In the current study, MB concentration at which the maximum value of q is reached depends on the value of interaction energy between each membrane and $M{B}^{+}O{H}^{-}{\left({H}_{2}O\right)}_{n}$. The higher it is, the lower is MB concentration. The range of MB concentration at which the maximum value is reached is between 250 and 300 mg L^−1^.

**Figure 8:**
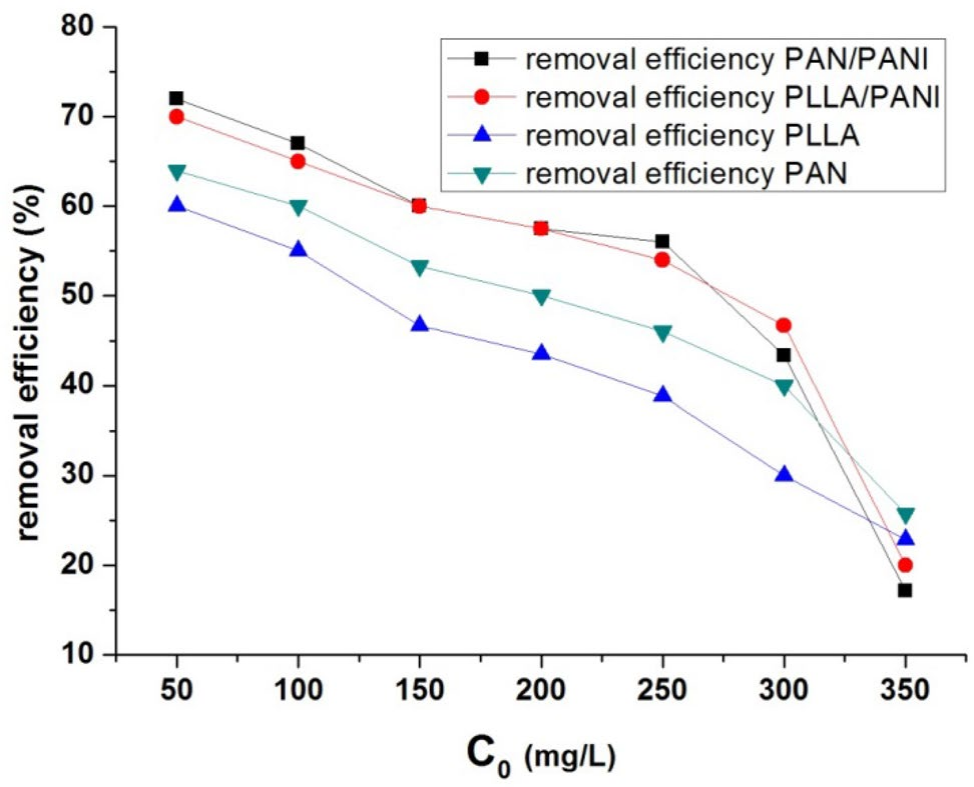
Effect of initial MB concentration on the removal efficiency.

The removal efficiencies of the all prepared membranes decrease with the increase of the initial concentration of methylene blue and this is due to greater number of MB molecules that needs more active sites to be adsorbed (Fig. 8)^30^.

In other words, as the initial concentration dye increases, the ratio of the available adsorption sites on the adsorbents to the total MB molecules number in the solution becomes smaller at constant adsorption dosage. Hence, a decrease in removal efficiency occurs.

From the results of adsorption capacity and removal efficiency, it is clear that PANI coating on PLLA and PAN membranes has a beneficial effect on adsorption performance of the membranes. The superior performance of PLLA/PANI and PAN/PANI membranes compared with non-coated ones could be attributed to their better wettability and stronger interactions between MB and the functional groups on the surface in comparison with PLLA and PAN membranes, respectively. These two factors play apparently a dominant role in the adsorption process in spite of the relatively decreased porosity and specific surface due to PANI deposition on PLLA and PAN. The slightly better adsorption performance of PAN/PANI membrane than PLLA/PANI membrane could be related to its higher specific surface area.

#### Effect of pH on adsorption

**Figure 9:**
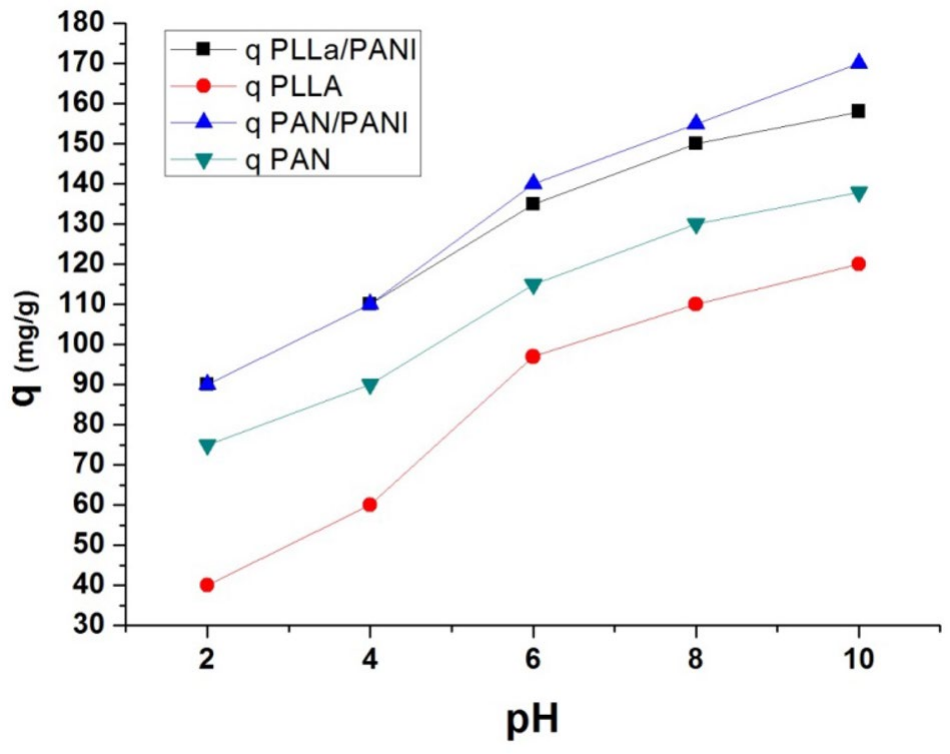
Effect of pH values on the adsorption capacity of the four membranes.

Methylene blue bears in its water solutions a positive charge, as a cationic dye. Thus, the adsorption on the membrane surface is largely affected by the surface membrane charge and it is also affected by the pH of the medium. The curves of Fig. 9 shows the behavior of adsorption capacity at different pH values, from strong acid to strong alkaline (pH values: 2, 4, 6, 8, and 10). The following parameters were fixed during the experiments at different pH values: methylene blue concentration is of 250 mg L^−1^ at 20 °C and adsorbent dosage m/v = 1/1 g L^−1^.

As shown, the adsorption capacity increases with pH increase. This is due to negative charges of the functional groups on the prepared membranes. In addition, the competition between protons and MB molecules becomes lower at high pH values.

#### Effect of temperature on adsorption

To look into temperature effect on the adsorption process of the blue dye on the four types of membranes, dye concentration has been fixed at 250 mg L^−1^, the adsorption dosage ratio (m/v) is at 1/1 g L^−1^, and pH at 6. Then, several adsorption experiments were conducted at different temperatures: 20, 30, 40, 50 °C.

**Figure 10:**
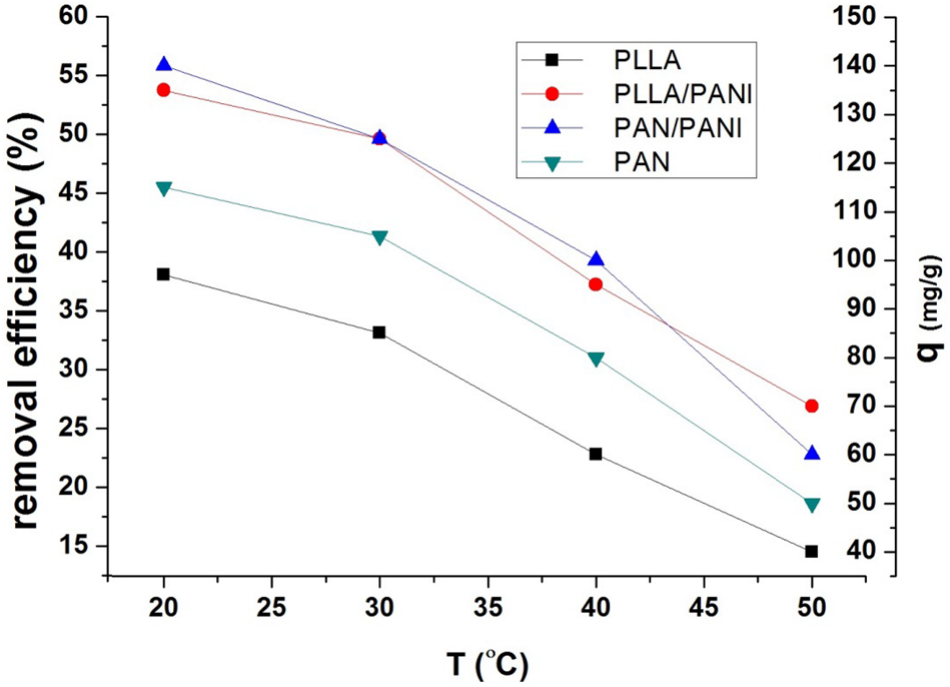
Effect of temperature change on the adsorption capacity and removal efficiency.

Figure 10 shows the effect of temperature on adsorption capacity and the removal efficiency by the different membranes, respectively. The adsorption capacity decreases as the temperature increases. This indicates that the interactions between the positive charges of the dye and the functional groups on the prepared membranes is lower at high temperatures, and this is due to the fact that the heat increases the kinetic energy of the molecules, so they cannot be adsorbed due to higher energy needed for the adsorption process to occur.

**Figure 11:**
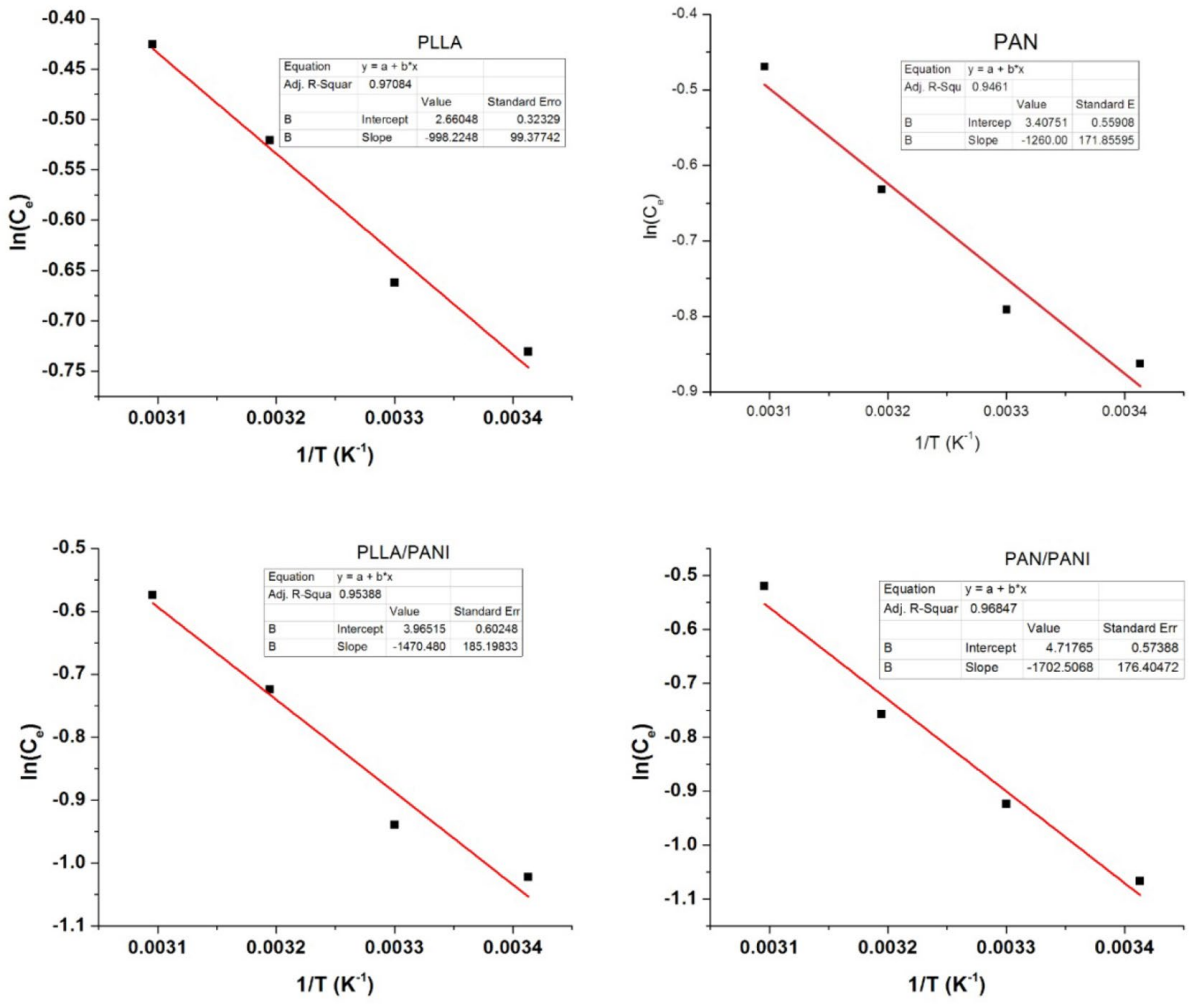
Plots of ln(Ce) vs 1/T for all prepared membranes.

To have deeper understanding of the nature of the adsorption process, the isosteric heat of adsorption, $\Delta H_{{{\text{ads}}}}$, was estimated using Clausis–Clapeyron equation, Eq. (5) ^37^

5 $${\left(\frac{\partial ln({C}_{e})}{\partial \frac{1}{T}}\right)}_{{n}_{a}}=\frac{\Delta {H}_{ads}}{R}$$

where $R$ is the gas constant, and ${n}_{a}$ is a given amount of adsorbed pollutant. The isosteric heat of adsorption of each membrane is estimated by plotting $\ln (C_{e} )$ vs. ${1 \mathord{\left/ {\vphantom {1 T}} \right. \kern-\nulldelimiterspace} T}$ as shown in Fig. 11^37^. The results are listed in Table 5. The negative values of all the calculated heat of adsorption of the four membranes suggest that the adsorption process is exothermic. The higher the absolute value of the isosteric heat, the stronger is the interaction between MB and the membrane. According to the listed values, the strongest interaction between adsorbate–adsorbent is for PAN/PANI membrane, then for PLLA/PANI membrane, after that for PAN membrane, and finally comes PLLA membrane. This result is in perfect match with the experimental results that show that the adsorption performance of the coated membranes is better than non-coated ones. The adsorption performance decreases as follows: PAN/PANI, PLLA/PANI, PAN, and PLLA.

**Table 5:** Isosteric heat of adsorption of the four prepared membranes.

Membrane	Isosteric heat (kJ mol^−1^)
PLLA	− 8.30
PLLA/PANI	− 12.23
PAN	− 10.48
PAN/PANI	− 14.15

#### Effect of adsorbent dosage (m/v)

**Figure 12:**
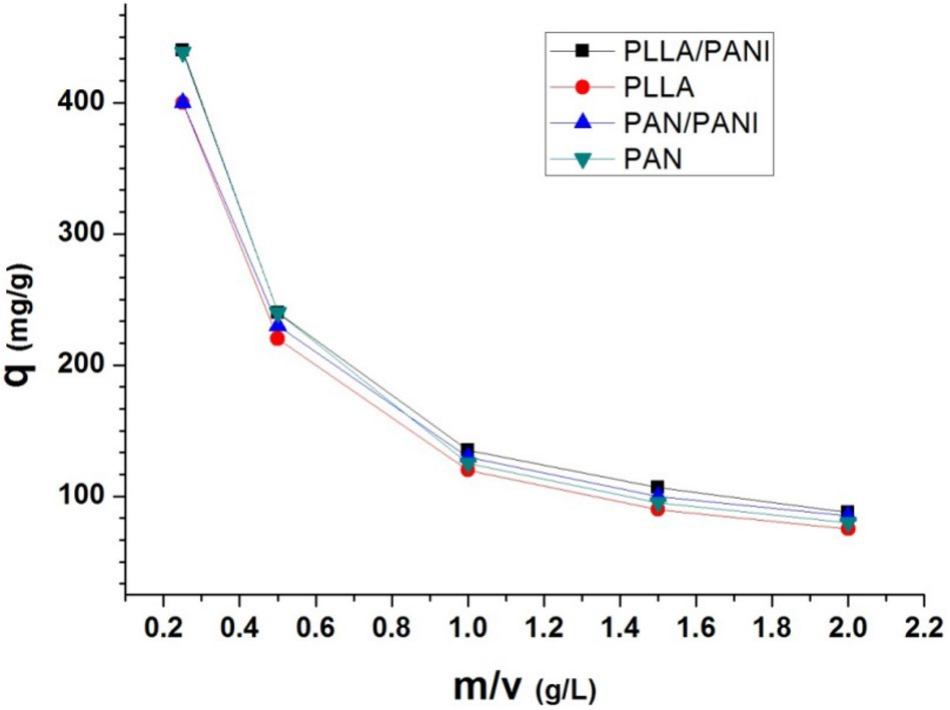
Effect of the m/v ratio on the adsorption capacity.

**Figure 13:**
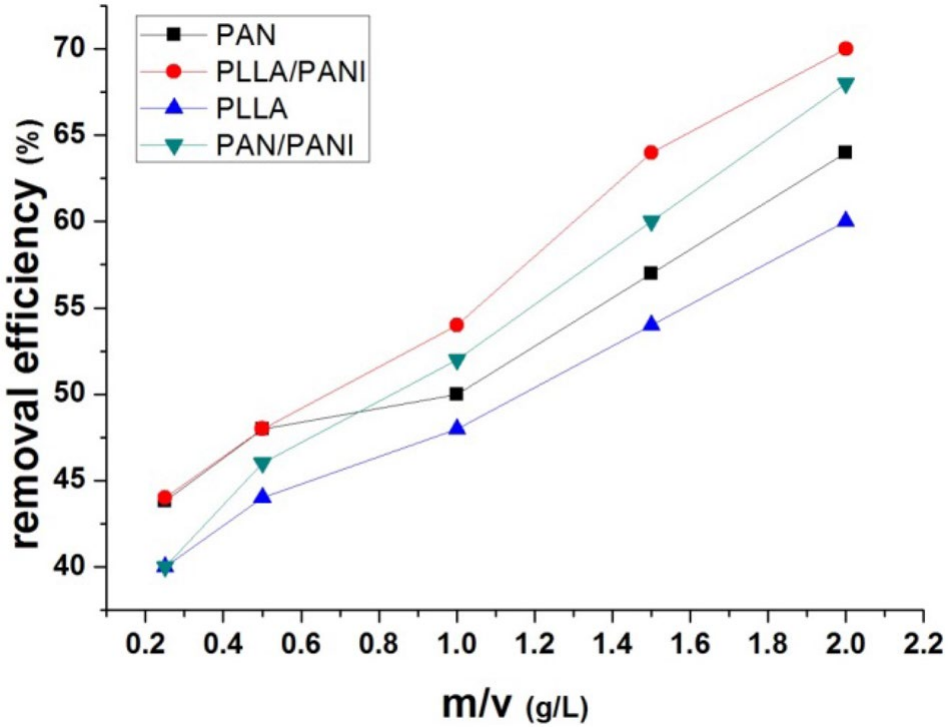
Effect of the m/v ratio on the removal efficiency.

The effect of (m/v) ratio of membrane weight to volume of adsorbate solution, adsorbent dosage, was studied while keeping constant the following parameters: concentration of methylene blue (250 mg L^−1^), pH = 6 and temperature at 20 °C for the four prepared membranes. Then, the ratio (m/v) was increased from 0.25 to 2 g L^−1^. The adsorption capacity and removal efficiency are shown in Figs. 12 and 13, respectively. The adsorption capacity decreases when the ratio increases. When the ratio (m/v) increases, at constant concentration of the dye, the adsorption capacity of adsorbent available is not fully utilized at a higher adsorbent dosage in comparison to lower adsorbent dosage. Moreover, the tension between liquid and solid surfaces also increases. As a result, the driving force of the mass transfer decreases and this leads to lower adsorption capacity. On the other hand, the increase in removal efficiency can be related to more active adsorption sites on the adsorbent surface at higher (m/v) values^30^.

The removal efficiency increases with the increase of the dosage ratio. This can be explained by the availability of a greater number of adsorption sites, Fig. 13.

In all the above-mentioned figures, the membrane adsorption performance decreases as discussed in “Effect of methylene blue concentration” according to the following order: PLLA/PANI, PAN/PANI, PAN, and PLLA.

#### Effect of contact time

**Figure 14:**
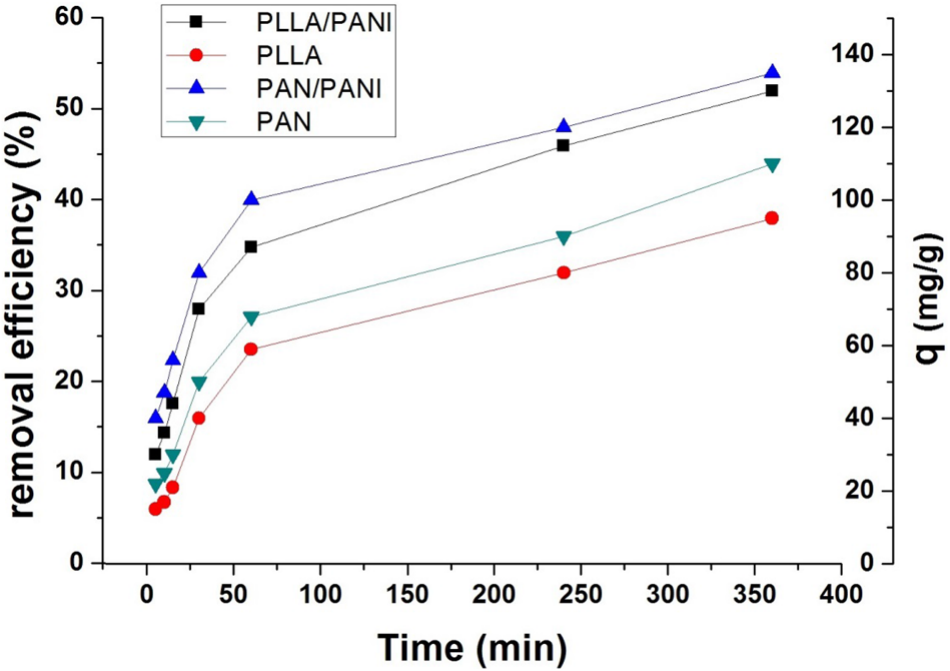
Effect of contact time on the adsorption capacity and removal efficiency.

The contact time effect was studied at the following constant parameters: adsorbent dosage ratio m/v = 1 g L^−1^, concentration of MB (250 mg L^−1^), pH = 6 and the temperature were kept at 20 °C for the four prepared membranes. Figure 14 exhibits the effect of contact time on the removal efficiency and adsorption capacity evaluated for the four membranes. As shown, there is a fast uptake in the first 60 min, then the curves tend towards a plateau when the available sites become occupied. PANI-coated membranes show faster MB uptake than the non-coated ones. The slightly superior performance of PAN/PANI membrane in comparison of PLLA/PANI membrane is linked to the stronger interaction between MB and PAN/PANI membrane, which has already been proved by its highest value of isosteric heat.

### Adsorption isotherms study

Different models that fit the adsorption isotherm curves are described in the literature^38–40^. They are useful to optimize the use of the membranes. In the current study, the adsorption of methylene blue solution by the prepared membranes was characterized using Langmuir and Freundlich models^38–40^.

Langmuir and Freundlich isotherm models are common isotherms models used to determine the affinity between the adsorbent and adsorbate and they give ideas about the adsorption mechanism. In Langmuir model, the adsorption is considered as a mono-layer process at distinct and energy-equivalent sites, whereas in Freundlich model, adsorption is considered as a multilayered process in which the adsorption sites on the surface and at the interlayers are heterogeneous and non-equivalent. The Langmuir and Freundlich models are described by the following Eqs. (6 and 7), respectively^39,41^:

6 $$\frac{{C}_{eq}}{q}=\frac{1}{b{Q}_{max}}+\frac{{C}_{eq}}{{Q}_{max}}$$

7 $$log q=log {K}_{F}+\frac{1}{n}log {C}_{eq}$$

where q is the equilibrium adsorption capacity at certain MB concentration (mg g^−1^), C_eq_ is MB equilibrium concentration (mg L^−1^), b is Langmuir constant, Q_max_ refers to maximum adsorption capacity (mg g^−1^), K_F_ is Freundlich constant and n is a heterogeneity index.

**Figure 15:**
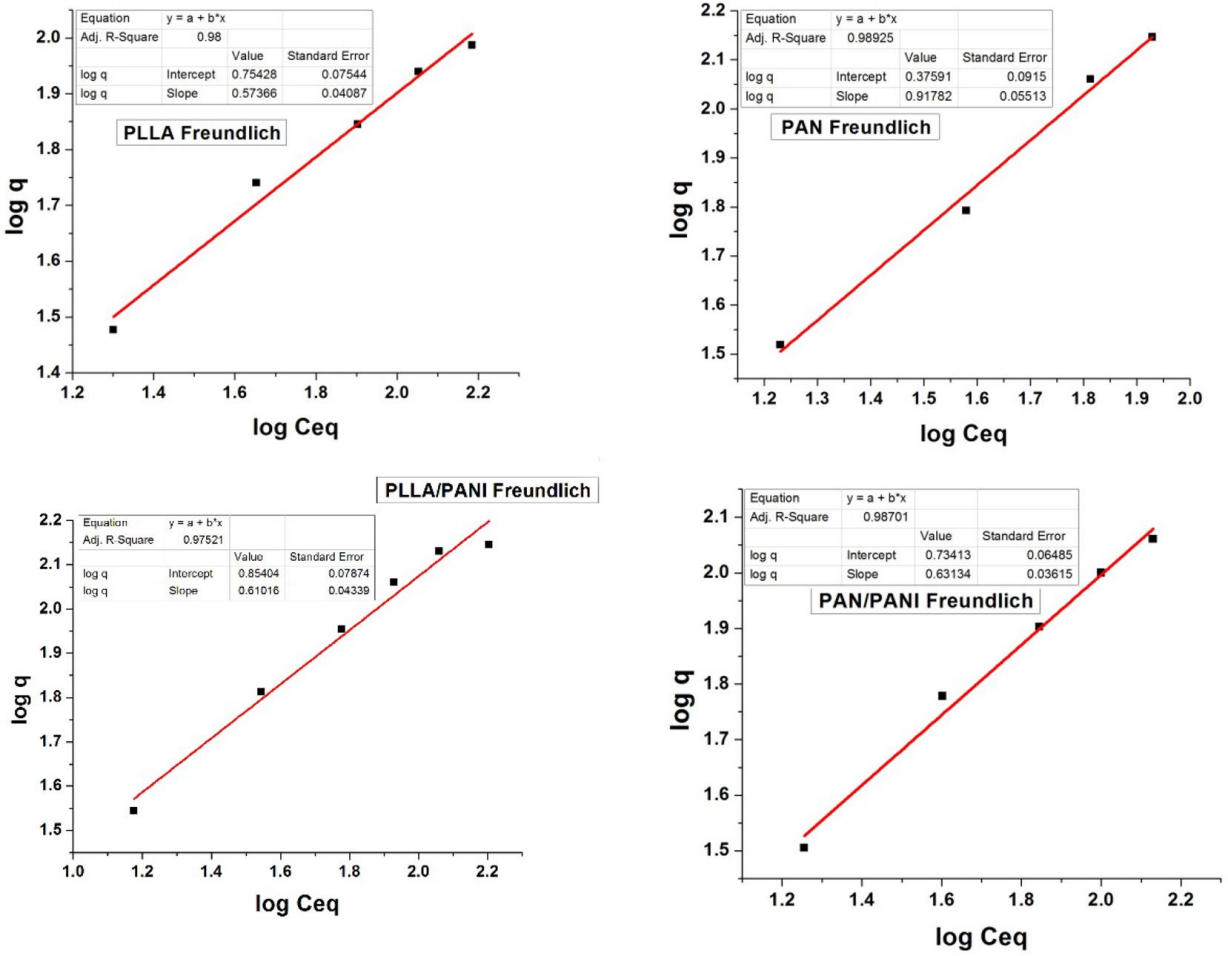
Plots of experimental data fitting according to Freundlich isotherms.

**Figure 16:**
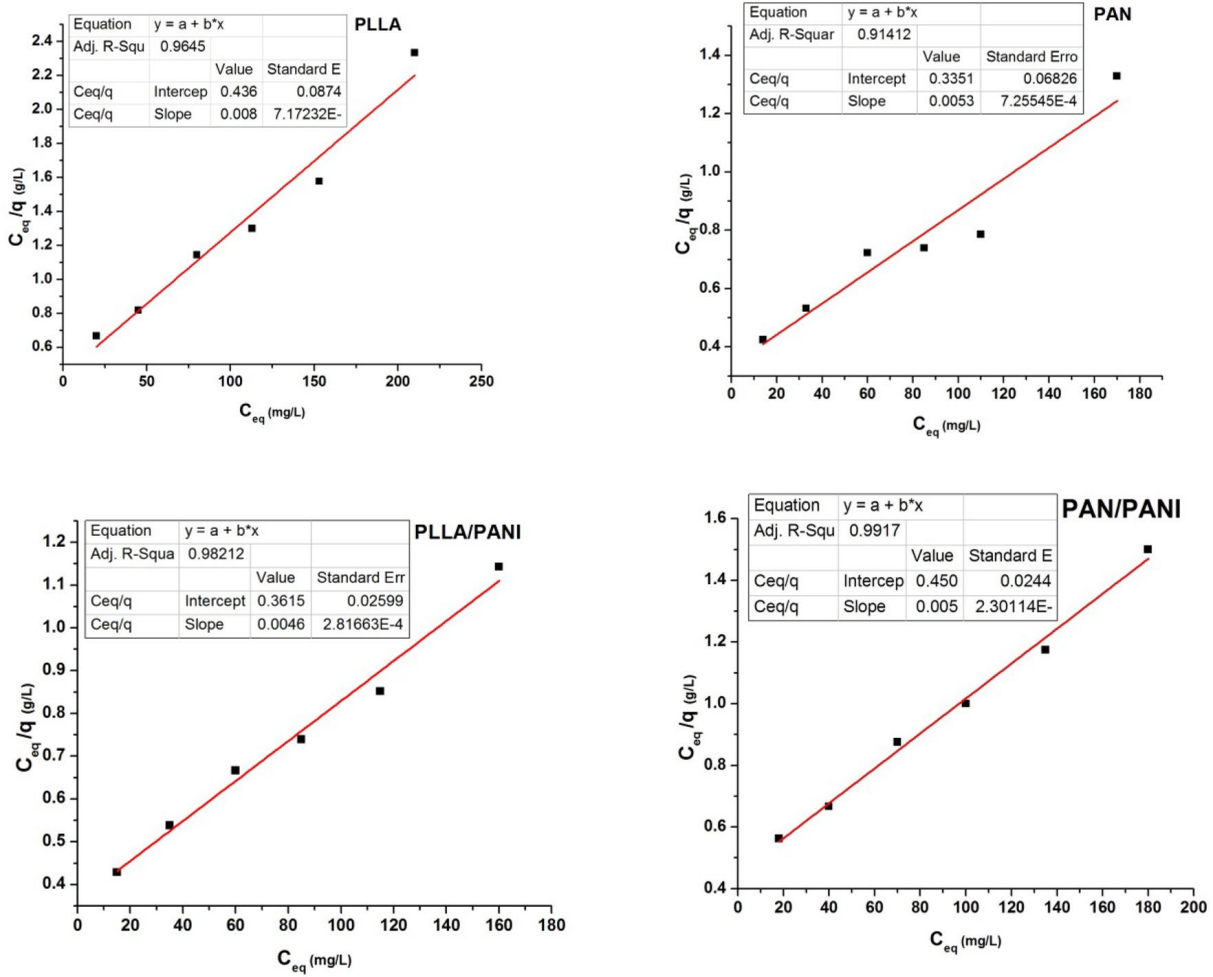
Plots of experimental data fitting according to Langmuir isotherms.

The results of plotting the curves of Freundlich and Langmuir isotherms are presented in Figs. 15 and 16, respectively. The estimated values of the constants of Freundlich and Langmuir isotherms are listed in Tables 6 and 7, respectively.

**Table 6:** Parameter values of Freundlich isotherms for MB adsorption on each of the four membranes.

Freundlich isotherm			
Membrane	*K*_*F*_	n	R^2^
PLLA	5.6791	1.741319	0.9800
PAN	5.42163	1.58393	0.98925
PLLA/PANI	29.8098	7.0407	0.91139
PAN/PANI	2.37634	1.08953	0.97521

**Table 7:** Parameter values of Langmuir isotherms for MB adsorption on each of the four membranes.

Langmuir isotherm				
Membrane	Q_m_ (mg g^−1^)	b (L mg^−1^)	R^2^	$\Delta {\text{G}}^{0} {\text{ kJ mol}}^{ - 1}$
PLLA	74.51	0.07038	0.9645	− 24.413
PAN	134.77	0.02638	0.91412	− 33.240
PLLA/PANI	239.23	0.01506	0.98212	− 20.657
PAN/PANI	398.40	0.005640	0.9917	− 18.264

From Tables 6 and 7, one can conclude that adsorption of MB on PLLA and PAN membranes of is slightly better described by Freundlich isotherms, whereas the adsorption of MB on PANI/PLLA and PANI/PAN is better described by Langmuir model. However, for the four types of membranes both Langmuir and Freundlich isotherms might to a certain extent describe the same set of adsorption data at the studied concentration range of MB. This could be linked to (1) the low range of MB concentrations in comparison to relatively large adsorption capacity of the membranes. As a result, both isotherm equations approach a linear form.

### Kinetic study of the adsorption process

Adsorption kinetics allows following the change of adsorption capacity over time. The experimental values of adsorption capacity were recorded during the time, using a solution of methylene blue at an initial concentration of 250 mg L^−1^. The kinetic data were fitted using first and second order kinetic models described by the following Eqs. (9 and 10):

9 $$ \log (q_{e} - q_{t} ) = - \frac{{k_{1} t}}{2.303} + \log q_{e} $$

10 $$ \frac{t}{{q_{t} }} = \frac{1}{{k_{2} .q_{e}^{2} }} + \frac{t}{{q_{e} }} $$

where $q_{e}$ is the adsorption capacity after 24 h, $q_{t}$ is the adsorption capacity at a definite immersing time, $k_{1}$ (min^−1^) and $k_{2}$ (mg^−1^ g min^−1^) are the rate constants for first and second order kinetics model, respectively.

**Figure 17:**
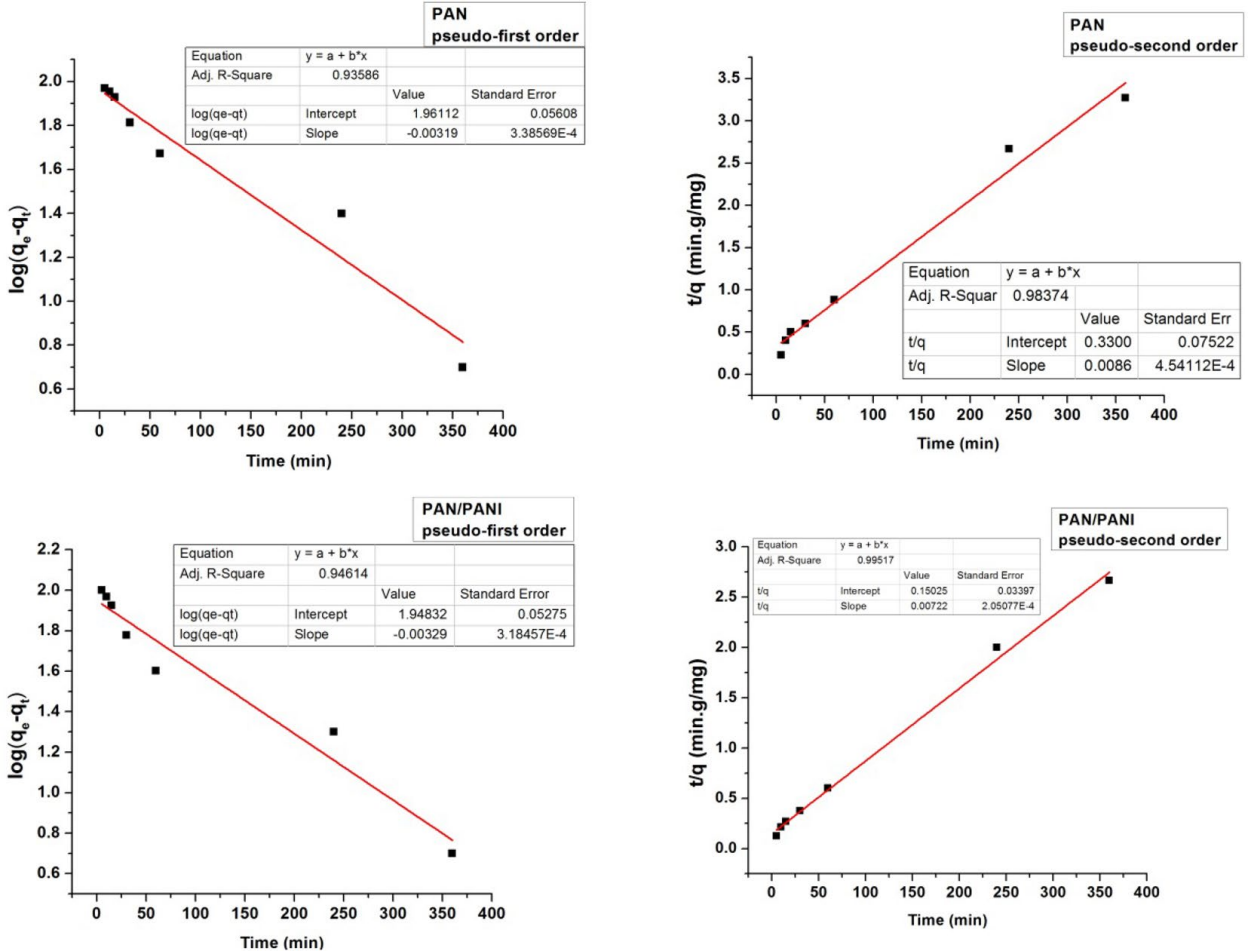
Plots of the pseudo-first and pseudo second orders kinetics related to the adsorption of MB on PAN & PAN/PANI nanofibers membranes.

**Figure 18:**
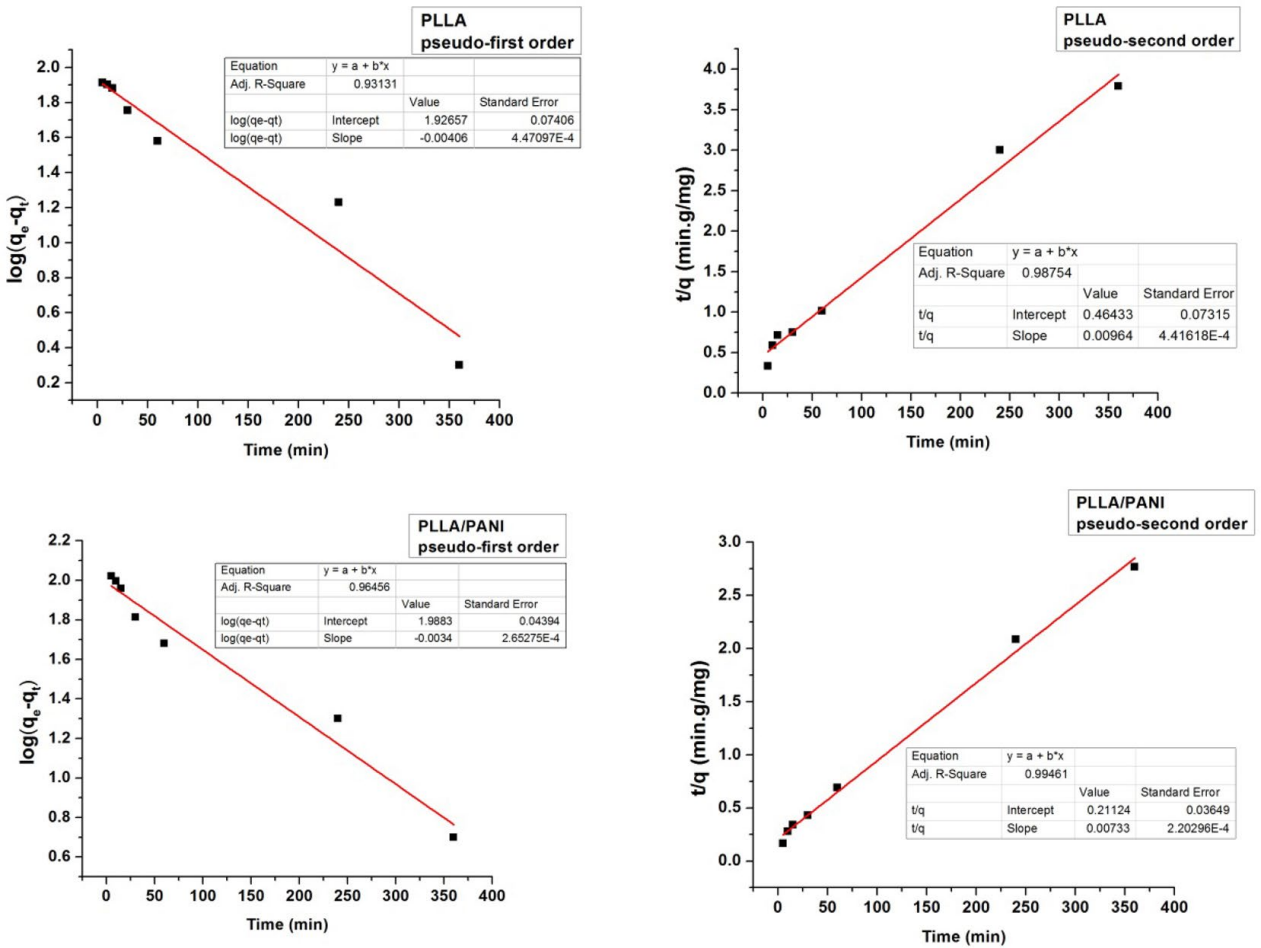
Plots of the pseudo-first and pseudo second orders kinetics related to the adsorption of MB on PLLA & PLLA/PANI nanofibers membranes.

All the kinetic parameters were determined from the intercepts and the slopes of respective plots, Figs. 17 and 18.

From the Figs. 17 and 18 and according to correlation factor of the fitted lines (R^2^ values), one can conclude that the second-order kinetic model better described the adsorption process for the four membranes, Table 8. The applicability of the pseudo-second order model indicates that the chemisorption is dominant when MB is adsorbed by the membranes.

**Table 8:** Adsorption kinetics parameters for the MB adsorption on nanofibers membranes.

Membrane type	q_e2_ (mg g^−1^) second order	q_exp_ (mg g^−1^)	k_2_ (mg^−1^ g min^−1^)	R^2^ (first order)	R^2^ (second order)
PLLA	103.73	97	0.00020014	0.93131	0.98754
PAN	115.34	115	0.00022778	0.93586	0.98374
PLLA/PANI	136.42	135	0.00025435	0.96456	0.99461
PAN/PANI	138.50	140	0.00034694	0.94614	0.99517

To gain deeper insight into the adsorption performance of the prepared membranes, their maximum molar adsorption capacity is compared with those of other electrospun polymer polymers in Table 9. Since different adsorbents are reported in the literature for different electrospun polymer membranes, maximum molar adsorption capacity is more appropriate than mass maximum adsorption capacity to assess the adsorption performance of each membrane towards pollutants. According to the results of Table 9, one can readily infer that the current PANI-coated membranes exhibit excellent adsorption performance (Q_max_ equals 0.747 and 1.244 mmol g^−1^ for PLLA/PANI and PAN/PANI membranes, respectively) in comparison with other electrospun membranes.

**Table 9:** Recapitulative table of Qmax of some polymer membranes with the current membranes in the study.

Sample	Pollutant	Q_max_ (mg g^−1^)	Q_max_ (mmol g^−1^)
PLLA/PANI (current work)	Methylene Blue	239	0.747
PAN/PANI (current work)	Methylene Blue	398	1.244
PANI/PS^27^	(Hg(II)	148	0.738
	Cd(II)	124	1.104
	Pb(II)	312	1.506
	Cr(VI)	58	0.299
	Cu(II))	171	2.691
TiO_2_-PANI/PAN^28^	black dye	130	0.131
pTSA-PANI/PLLA^29^	Methyl orange	400	1.222
keratin nanofibers^30^	Methylene Blue	170	0.531
Polydopamine-coated electrospun poly(vinyl alcohol)/poly(acrylic acid)^42^	Methylene Blue	180	0.563
PANI/clinoptilolite^43^	Acid violet 90	250	0.265

### Re-use of prepared membranes

**Figure 19:**
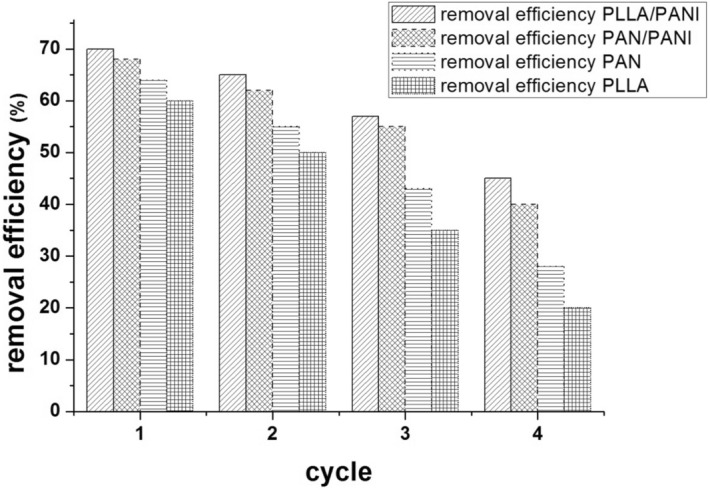
Reuse of the four membranes.

For economic and environmental purposes, the ability of the membranes to be reused has to be investigated. The four prepared membranes, initially subjected to a 250-ppm methylene blue adsorption experiment, were immersed in 0.1 HCl solution for 30 min and then washed abundantly with water. Then, each of the regenerated membrane was tested to be re-used several times by immersing it into methylene blue solution, (at MB concentration of 250 mg L^−1^, 20 °C, pH = 6, and maintaining the adsorbent dosage at the ratio m/v = 1/1 g L^−1^) and then re-generating it. The removal efficiency was measured after each time of immersion. The results for the four membranes, depicted in the diagram, Fig. 19, show the possibility of re-using the membranes several times. The coated membranes PAN/PANI and PLLA/PANI show superior performance, since they kept about 59% and 63%, respectively of their removal efficiency after a total of four cycles of adsorption–desorption (Counter 44% and 48% for PAN and PLLA membranes, respectively). The excellent performance of PANI coated membrane could be linked to easier desorption of MB at low pH, owing to repulsive electrostatic forces between positively charged emeraldine salt of PANI and the cationic pollutant MB.

## Conclusions

In this work, MB adsorption on four types of membranes, PLLA, PAN, PLLA/PANI and PAN/PANI have been examined. FTIR and UV–Vis spectroscopy indicated the successful PANI coating on PLLA and PAN membranes, whereas SEM micrographs showed the fibrous morphology of the membranes. The superior adsorption characteristics obtained by combining the conductive PANI coating with the porous electrospun nanofibrous membranes of PLLA and PAN were linked to (1) enhanced membrane wetting properties and (2) promoted electrostatic interactions between the adsorbent and the adsorbate. The strength of the interaction between MB and the membranes was evaluated by estimating the isosteric heat of adsorption for each membrane. The results showed stronger interactions between MB and PANI-coated membranes in comparison with non-coated ones. The highest absolute value of isosteric heat for PAN/PANI membrane is linked to its higher specific area in comparison to PLLA/PANI membrane. DC-conductivities of coated membranes were correlated to MB concentrations, which suggests the potentiality of the use of such mats as MB concentration sensors. The reusability of the four membranes was tested and PANI-coated membranes exhibited the best performance, suggesting the beneficial role of coating the membranes with PANI. All the above-mentioned features render PLLA/PANI and PAN/PANI promising membranes for water treatment.
